# Work-related stress amongst legal medical doctors: the need for systematic psychological support. An Italian perspective

**DOI:** 10.1093/fsr/owad018

**Published:** 2023-06-23

**Authors:** Sara Sablone, Matthew Groicher, Tamara Patrizia Fanco, Roberta Risola, Grazia M Violante, Mara Bellino, Valeria Lagona, Ignazio Grattagliano

**Affiliations:** Interdisciplinary Department of Medicine, Section of Forensic Medicine, University of Bari, Aldo Moro, Italy; Department of Education Science, Psychology and Communication, University of Bari, Aldo Moro, Italy; Department of Education Science, Psychology and Communication, University of Bari, Aldo Moro, Italy; Department of Education Science, Psychology and Communication, University of Bari, Aldo Moro, Italy; Department of Education Science, Psychology and Communication, University of Bari, Aldo Moro, Italy; Interdisciplinary Department of Medicine, Section of Forensic Medicine, University of Bari, Aldo Moro, Italy; Interdisciplinary Department of Medicine, Section of Forensic Medicine, University of Bari, Aldo Moro, Italy; Department of Education Science, Psychology and Communication, University of Bari, Aldo Moro, Italy

**Keywords:** forensic sciences, forensic practice, work-related stress, burnout, risk, psychological support, psychotherapy

## Abstract

Legal medical doctors (LMDs) expertise encompasses a wide range of responsibilities. Work-related stress amongst LMDs is an extremely relevant factor, which affects the quality of LMDs life and work. Whilst it is a better-known problem abroad, this issue is poorly debated in Italy, as demonstrated by this literature analysis. The aim of this paper is to better understand the main sources of stress in the practice of legal medicine in Italy, highlighting the need for systematic psychological support. The risks of work-related stress for the social and health professions are well known in literature. In Italy, however, due to a series of complex circumstances and cultural and research delays, forensic practitioners often seem to be excluded from these kinds of complex issues. The authors, after a series of considerations drawn from a review of the literature and from experience in the forensic and medical field, point out how work in this area entails risks for workers, on par with all others belonging to the helping and social-health professions. They conclude their contribution with a series of proposals for appropriate protocols to cope with such problems for workers in the forensic and medical field.

**Key points:**

- Work-related risks for the social and health professionals are widely recognized in the literature.
- Although legal medical doctors are often thought to be safe from these types of issues, significant risks exist for this category as well.
- It is important to create appropriate protocols to address outcomes for legal medical doctors.

## Introduction

Forensic professionals, who are often members of multidisciplinary evaluation teams, need to show intelligence, intuition, openness, and empathy to capture both the causes and the context of the diagnosis and to provide continuity from crime scene to courts of law from trauma/event to trial [1, 2].

According to the current regulatory framework established by the Italian Ministry of University and Research, physicians specialized in legal medicine, known as legal medical doctors (LMDs), can be counted amongst forensic professionals. In Italy, legal medicine is a field concerned with scientific and educational training activities of forensic medicine. Its specific areas of competence are social medicine, criminology, forensic psychopathology, forensic toxicology, deontology, medical ethics, and clinical bioethics [3].

LMDs are physicians with expertise in evaluating biological phenomena with a juridical interest. This title is gained after at least 4 years of specialization following a degree in Medicine and Surgery. Overall, LMDs place their knowledge of medicine and law at the service of the judicial authorities to enable the administration of justice.

Depending on the area of professional employment, LMDs may be frequently exposed, both directly and indirectly, to graphic and traumatic material whilst visiting crime scenes, as well as through secondary means, including victim accounts, photographs of crime scenes, digital material, physical evidence, and case notes [4]. Furthermore, they may often be in contact with corpses, human remains, and other potentially shocking scenes in the autopsy room or on crime scenes. LMDs may be involved in identifying the deceased, determining the causes and modalities of death, and communicating this information to the interested parties, including family members, law enforcement, and health and judicial authorities. Moreover, LMDs may intervene on the sites of mass disasters, or mass fatal incidents (MFIs), for the recognition of the victims, exposing these professionals to the risk of developing symptoms of acute stress disorder (ASD) and post-traumatic stress disorder (PTSD) [5]. Additionally, LMDs may play a main role in ensuring the continuity of care in healthcare institutions, by evaluating the victims of crime in the emergency department, but also by solving problems regarding patient consent to invasive medical treatments and procedures [6]. Therefore, LMDs employed in healthcare institutions could be responsible, even in emergencies, for documenting, collecting, and storing evidence of criminal offences that may be altered over time (such as photographs of bodies and clothes or other belongings of victims), and establishing the lawfulness of invasive medical acts on patients who are incapable of self-determination but in need of such treatments. LMDs may also be called to determine whether there has been malpractice, or to assess a medical colleague's professional liability for damage to patients, always keeping in mind the continuous updating of professional standards, good clinical practices, recommendations, and specific guidelines [7]. Therefore, LMDs’ professional assessments may concern various categories of patients (such as children, adults, inmates, abused people, inpatients, psychiatric, or incapacitated subjects, etc.) and the diverse casuistry requiring legal medical professionalism is paralleled by an emotional load specific to this job [8].

All these professional tasks are emotionally exhausting and can have detrimental effects on professionals’ attitudes, behaviour, outlook, and judgement on life or death [1].

In such high-pressure working environments, the quality of LMDs’ assessments can be compromised, thereby influencing the accuracy of decisions, the confidence levels of judgements, and the ability to document and interpret the conclusions of investigators, judges, and other stakeholders [9–11]. LMDs’ cumulative exposure to the numerous stressors their job entails may be subject to adverse effects, including PTSD and other conditions [4], which will be discussed in depth later. Previous research has focused on these conditions in first responders, such as police officers, who are at risk for symptoms of PTSD and depression, due to frequent exposure to disturbing facts in the line of duty [12]. Unfortunately, LMDs have been largely excluded from this research. Furthermore, there have been very few suggestions regarding treatment for first responders suffering from the psychological impacts of their professions, and even less research has been done regarding psychological support for forensic practitioners such as LMDs, medical examiners, or coroners. Although mental health needs for emergency workers have been identified, there are no data on supportive interventions for forensic professionals. This is highly concerning, considering some studies have found that over 9% of forensic practitioners show signs of being at risk for PTSD, with the most frequently reported symptoms being negative beliefs about oneself, others and the world, and hypervigilance [4].

This scholarly contribution strives to highlight that, despite the fact that LMDs are known to be exposed, both directly and indirectly, to various situations with a significant emotional impact that can lead to consequences on their mental health, their conditions of psychological distress are often underestimated or even ignored. This results in a lack of adequate support for this category of professional. In Italy, there are no defined and precise protocols to address the stress, tension, suffering, and risk of burnout facing LMDs engaged in their professional activities. Thus, it is abundantly clear that there is a need for systematic mental health support amongst professionals in the field of legal medicine, to better assess their stressful activities and reduce the risk of mental health problems. As the state of current literature stands, however, there is no consensus on best practices for providing this support. This paper aims to contribute to resolving this gap in the literature by advancing some recommendations for supporting LMDs, and potentially other forensic workers with similar working conditions, struggling with job-related mental health issues, as well as stimulating further investigation in this line of research.

## LMDs and the risk of stress-associated symptoms

Edwards [13] describes stress as a negative discrepancy between an individual’s perceived state and desired state, provided that the individual considers the presence of this discrepancy relevant. Earlier work by Dewe [14] suggests that definitions of stress should reflect its relationship to adaptive factors. The individual’s ability to cope with external pressure is dependent on a cognitive appraisal of the stressor and the coping strategies the person has available to them. Stress, therefore, is perceived in relation to an individual’s previous experience, as well as their success or failure in dealing with similar situations [14].

Stress, therefore, is a nonspecific reaction of the body to internal or external stimuli, which triggers adaptive or readjustment mechanisms in order to restore homeostasis. This stress can be either adequate to stimulate growth in the individual (eustress), or dysfunctional, chronic, or otherwise harmful to the individual (distress). Determining the difference between positive stress and negative stress is essential to guiding stress management and to promoting behaviours related to psychophysical wellbeing [15–20].

Distress generates negative consequences on personal, social, and business fronts. In a work context, dysfunctional stress occurs when there is an imbalance between the demands made of workers and the abilities and resources at their disposal to meet those demands [21]. Work-related stress can be caused by factors as diverse as the content of the work, possible inadequacy in the management of work organization and work environment, communication failures, and violence and aggression [21].

Individual factors can also influence the effects of distress on those working in the field of legal medicine. A person’s reaction to stress depends not only on the event itself but also on the subject’s emotional and physiological response. Unpredictable, uncontrollable, or threatening events can be perceived as highly stressful and the magnitude of stress generated can be influenced by the characteristics of the individual, those of the environment, or those of the event. Individual risk factors may include a history of physical or psychiatric illness, having experienced significant trauma in the past that is reactivated through victims’ experiences, secondary stressors such as excessive workload, or institutional policies that limit coping strategies [5].

Multiple studies have documented the stressful nature of forensic professions. Particularly stressful cases are those involving infant deaths, unpredictable or painful deaths, and deaths in which staff identify with the victim [22, 23]. The stressors most associated with psychological symptoms include exposure to accidental deaths of children and interaction with the families of the deceased. Forensic workers frequently identify with the circumstances that could lead to these accidental deaths [24]. Mass disasters and MFIs, which are characterized by an extensive number of casualties, have also been found to be highly stressful to those in occupations that are indirectly exposed to their effects [25]. Disaster workers report that identification with the dead and their families is particularly stressful and involves feelings of “It could have been me”, “It could have been my spouse”, or “It could have been my friend” [26]. Furthermore, stress associated with interacting with family members of the victims is a significant predictor of all types of psychological symptoms [27].

During court cases, LMDs called to perform examinations may be subject to pressure aimed at inducing them to align with one legal side over another, sometimes leading to threats, violence, or retaliation against the involved LMDs who do not comply with this pressure [28, 29]. Furthermore, the intensified scrutiny of forensic techniques and criticisms of their validity, being subject to cross-examination in court, and working in a culture that does not tolerate errors further exacerbates stress whilst working on these cases [7, 30–32]. Pressure from managers or supervisors, as well as case backlogs are also identified as factors that contribute to stress in the workplace, although these can be common across different occupations [33].

The level of emotional exhaustion seems to be higher in LMDs who also have a teaching activity, likely due to the additional stress caused by the responsibilities pertaining to this activity [1].

Finally, weekly working hours, frequency of medical shifts, time pressure, caseload size, inadequate funding/staff, personnel management, role conflicts, pressure from the authorities and concerned families, as well as personality traits (resistance to stress, tolerance of frustration, extraversion, neuroticism, attitudes to people or life, susceptibility, etc.) contribute to a wide range of effects at a professional and personal level [1, 6].

## Effects of stress and exposure to graphic material

As a consequence of the aforementioned stressors in their work environment, LMDs, and other forensic professionals are at risk of various mental health problems, including, but not limited to, PTSD, vicarious traumatization, secondary traumatic stress (STS), compassion fatigue (CF), and burnout [4].

PTSD is a psychiatric disorder that may occur in people who have experienced or witnessed a traumatic event, series of events, or set of circumstances. An individual may experience this as emotionally or physically harmful or life-threatening and may affect mental, physical, social, and/or spiritual wellbeing [34]. This condition is generally seen as concerning those who have directly experienced a traumatic event; however, it has been observed that experiencing extremely stressful events second hand can also have detrimental effects on mental health. Vicarious trauma (VT) is a term originally used to describe therapists’ reactions to traumatic material expressed by clients; however, it has been found that other professionals who experience traumatic content indirectly may also be susceptible [4]. It involves the disruption of beliefs regarding the self, others and the world that results from cumulative exposure to traumatic narratives and indirect exposure to trauma experiences. This condition should be distinguished from STS, also known as CF, which consists in the development of symptoms (exhaustion, intrusion, hypervigilance, and avoidance) often experienced by healthcare professionals working with people impacted by trauma or PTSD and their family members [27, 35]. Due to the nature of their profession, these conditions present a clear risk to LMDs, in addition to the possibility of developing PTSD and symptoms of job burnout [4], a condition of work stress found most frequently amongst those engaged in professions concerning the social-health area [36].

Multiple factors have been found to influence the development of mental health issues amongst those working in the field of legal medicine. For example, identification with the dead is associated with higher rates of PTSD, including acute and long-term symptoms of avoidance and somatization [26]. Identification is not only a risk factor for adverse outcomes, but also a mechanism by which exposure to the dead leads to symptoms in workers present at scenes of disasters, as LMDs may be. The literature highlights different types of identification with the deceased, for example identification with the deceased as oneself, identification with the deceased as a friend, and identification with the deceased as a family member. The different types of identification are not equally predictive of PTSD and other symptoms; this affirms a level of specificity in the identification process. For example, younger individuals tend to identify with the deceased as a friend and report greater correlation to the manifestation of symptoms [26].

The duration and intensity of exposure to a traumatic event also increases the risk of PTSD. Forensic professionals such as LMDs often have prolonged exposure to traumatic incidents, as they are involved throughout the identification of the victim, as opposed to recovery workers who are present for a much more limited time [37]. Research has also shown that the number of critical incidents experienced by forensic personnel is positively related to symptoms linked to post-traumatic stress, such as intrusive thoughts and avoidance [25]. Other mediating factors may include sociocultural context, policies governing the way work is carried out, available social resources, organizational characteristics, sex, and exposure to both direct and indirect trauma [38].

## Providing support to trauma-exposed LMDs

Resilience is the property of materials to withstand impact without breaking. In humans, this means adapting and overcoming difficult, sometimes traumatic experiences through mental, emotional, and behavioural flexibility [39]. Physical fatigue, emotional duress, and the need to work often at the limits of their abilities put LMDs to the test on a regular basis. Faced with stressful situations such as this, LMDs and other forensic professionals may experience various effects on their mental health that, if not properly recognized and managed, may worsen over time. By implementing systematic psychological support for these professionals it may be possible to contribute to LMDs’ capacity for resilience, and therefore their ability to adapt and perform at their best in the highly stressful, yet essential career they have chosen.

How to support personnel who may be suffering from stress-related mental health issues is still a controversial topic. Whilst the literature on trauma-exposed forensic science employees is growing, a focus on individuals in forensic science disciplines is needed so that healthy interventions can be implemented, and organizational structures can be adapted to support these essential workers. Necessary interventions may be implemented at both the organizational and the individual level, in order to address systemic problems rooted in the day-to-day workings of organizations as well as specific cases of individuals suffering from mental health issues due to workplace stress.

### Organizational level strategies

The main theme emerging from the literature is the need for strong social support, both from peers and, especially, from leaders. Support from leaders has even been shown to be more important than support from friends or family and it is related to lower levels of psychological distress [40]. Furthermore, it has been proved that organizational support is associated with reduced levels of STS and burnout [41]. Organizational strategies such as flexible planning, effective communication with supervisors, and educational interventions to help employees recognize and relieve stress have all been found to be protective against psychological distress [42].

Therefore, it is vital that LMDs have access to, and be encouraged to seek help within the company, by meeting with the human resources manager and the occupational physician (competent doctor), who carries out health surveillance in the company. In Italy, both figures are appointed by the Legislative Decree no. 81, approved on 4 April 2008 [43] in order to supervise work risks and health effects and to identify and implement prevention and mitigation actions. The above mentioned Decree designed all the regulations on health and safety in the workplace. In this context, safety in the workplace must be understood as a set of interventions that must be adopted to protect the health of workers whilst carrying out their activities. The largest change promoted by this Decree is that the consolidated text on health and safety at work shifts its focus to prevention, by introducing obligatory preventive assessment of the risks present in the company. Following this assessment, it is necessary to plan and implement actions to improve safety and health in the workplace.

Another solution is to seek help from a psychotherapist in order to support workers in building the tools to cope with distress and regaining the energy to defend themselves. Work discomfort is more likely to occur in a context where dialogue is impossible, so it is important to re-establish dialogue in the business context to prevent such episodes [44]. Psychologists are the more appropriate figures who can help to re-establish dialogue in the business context. It is also of paramount importance that managers and supervisors are trained in best practices for taking care of their employees, as well as recognizing warning signs of mental health problems and implementing proactive and preventive strategies to safeguard their workers.

A commonly prescribed strategy is psychological debriefing, especially after critical events or MFIs. The goal of this strategy, generally conducted by mental health professionals soon after the event, is to allow employees to discuss the event and their feelings towards it in order to prevent the development of PTSD and other stress symptoms. However, a concerning amount of research shows that debriefing is ineffective and can even inhibit recovery in some cases [45]. Despite these findings, debriefing shortly after a traumatic event continues to be a widely used intervention. Since most people seem to make a full return to normal within the months after a traumatic event, it has been suggested that managers and supervisors should be trained to recognize symptoms of trauma-related stress in order to identify at-risk employees and recommend further treatment if necessary [45].

The Office for Victims of Crime has developed the “Vicarious Trauma Toolkit” to mitigate the potentially negative effects of exposure to trauma. This toolkit includes scorecards and protocols to assess employees' ability to cope with occupational exposure to trauma and develop an action plan [46].

With respect to job burnout, several prevention strategies are suggested on different levels, including staff and management development, job and role changes, better management of problem solving at the organizational level and decision-making moments [47]. As far as staff development is concerned, the authors suggest it may be possible to reduce the demands placed on professionals by setting more realistic goals, encouraging practitioners to aspire to goals that can lead to rewards, providing them with training opportunities, teaching them better time management, helping them to use feedback and control mechanisms in their work, preparing them for possible frustrations they may face in their profession, providing periodic Burnout Checks along with counselling services, and encouraging the development of sharing groups. With respect to goals, the authors suggest to make them clear and compatible with each other, so that employees can easily understand and achieve them, thus achieving gratification. Conflict situations, on the other hand, can be managed by creating formal group mechanisms for problem and conflict resolution, organizing training on these processes, and encouraging staff autonomy and participation in decision-making processes.

### Individual level strategies

In addition to the positive impact of organizational interventions, it is vital that LMDs receive education in effective coping strategies, allowing them to better manage stressful and potentially traumatic situations. These coping strategies should be seeking social support or discussing troubling events with coworkers, supervisors, loved ones, or even mental-health professionals, instead of giving up, drinking, or getting angry, which have been associated with an increased risk to develop PTSD symptoms [48].

Limited literature shows cognitive and emotional coping for forensic mental health personnel. Although forensic professionals have strategies to cope with their affective responses during exposure to death (for example, distancing or taking breaks), particular cases or MFIs can overwhelm existing coping resources [5]. Frequently employed coping strategies include emotional distancing (isolating oneself from the emotional experience of a given situation), and the use of humour [4]. For example, professionals in the area of sexual violence often use humour, light-hearted, and gallows, to counter the effects of work on their wellbeing. Gallows humour is defined as “humour that makes fun of a life threatening, disastrous, or terrifying situation” and it is used as a coping strategy by social workers, journalists, police officers, soldiers, and crime scene investigators [49]. However, there is little evidence to support the effectiveness of the use of humour in reducing symptoms of traumatic stress. Indeed, the excessive use of humour turns out to be harmful in the attempt to suppress frustrations and negative emotions [50]. An alarming finding from a 2019 study showed that only 18% of a sample of crime scene investigators reported talking to someone about a stressful work event as a frequently used coping strategy [48]. Moreover, there appear to be mixed findings regarding the frequency with which forensic employees seek professional help for mental health issues, though a 2003 study found that nearly a quarter of their sample reported seeking mental health consultation [27].

Noteworthy positive coping strategies include participating in activities such as exercise, prayer, meditation, and mindfulness [4]. A mindfulness-based resilience training course on dispositional mindfulness has been shown to be effective [51], whilst a study by Jeanguenat and Dror [7] shows how the use of mindfulness, awareness, relaxation, and reflection in a work environment can have a positive impact on decision-making. Furthermore, awareness and commitment to one's spirituality have both been shown to have a positive effect on those with VT or other forms of trauma. This type of self-care can be vital for those who have internalized a victim's trauma [7].

When coping strategies are insufficient and forensic professionals require assistance, what is the best way to intervene? Research in this field is severely lacking and tends to focus on professional figures such as first responders or medical doctors who frequently experience traumatic scenes first hand. Despite this, some general guidelines can be extracted from the existing literature.

Several strategies can be used to deal with PTSD symptoms, for example, for physical symptoms, relaxation exercises, mindfulness, or the use of anti-anxiety drugs can be helpful. For intrusive symptoms, Cognitive Behavioural Therapy treatments, Eye Movement Desensitization and Reprocessing, and the use of antidepressant drugs have been shown to have positive results. Avoidant symptoms can be coped with by talking about the event to gradually relive and process it [52].

Actively modifying the perception of events can not only allow better insight into them, but also a better management of reactions as the events occur, and consequently limit distress and dissociative symptoms [5]. To this end, interventions that include mindfulness and counselling could be useful for those suffering from VT or other forms of trauma [4].

An additional factor that appears to be crucial in managing traumatic stress is the awareness of perceived control. The perception of exerting some level of control over a situation allows people to better adapt to stressful situations and can be a protective factor against trauma-related disturbances [25]. Structured trauma counselling meetings can be implemented for those in need, whilst at an institutional level management can support interventions that improve morale and team relationships. All these examples have a similar focus of promoting the perception of control [25].

Considering the type of critical event and the specific situation, it is possible to introduce interventions with different approaches. If immediate preliminary support treatment is required, Psychological First Aid can be used to reduce stress symptoms [5]. Moreover, it has been proved that peer interaction can provide comfort by debriefing amongst colleagues with similar experiences [53]. For example, one suggestion would be to use the Assaultive Staff Action Programme, which is a voluntary, peer-support programme that can be used for this purpose [52].

## Discussion and conclusions

There are multiple professional strategies that could be made available to LMDs, such as supportive supervision, development, and training opportunities for stress management, debriefing opportunities, and greater level of flexibility at an organizational level. Unfortunately, organizations do not always invest enough resources in the wellbeing and health of employees. In Italy, satisfying employees’ needs is still frequently considered as a cost rather than an investment for employers. This is coupled with the idea that employees’ wellbeing is limited to their physical health, or the absence of disease, without taking into account their psychological, emotional, and relational health, all of which are equally important [54]. It is a well-documented fact that a “healthy” organization is also a more productive and efficient organization. But organizational health starts from that of the employees that make up its parts. Research conducted by BVA Doxa in 2021 found that nearly 50% of workers in Italy reported symptoms of work-related anxiety and insomnia, whilst 80% of respondents showed at least one symptom of burnout (feelings of exhaustion, reduced efficiency, mental detachment, and cynicism). Furthermore, 40% of the sample indicated they do not feel able to discuss their mental health related problems in the workplace. This lack of organizational support has led to frequent absenteeism and even workplace dropout by professionals who are seeking to preserve their mental health [55]. In conclusion, there are multiple stress-causing risk factors in work settings, some of which originate from the organizational context and others from the nature of forensic professions such as that of LMDs. Amongst the former are poor communication, low levels of support for problem solving and/or personal development, inefficient organizational goal setting, role ambiguity and conflict, insufficient promotions, job insecurity, limited relationships with superiors, interpersonal conflict, conflicting demands between family and work, and others. The latter include physical working conditions, working around the deceased, interacting with victims and/or victims’ families, exposure to graphic or violent material, and others. Given the stressful and traumatic nature of the activities in which LMDs are involved and the impact on the health and psychophysical wellbeing of the person, their family members and their context, immediate and structured interventions are necessary in Italian medical-legal contexts for the management of the risk and stress and, subsequently, treatment and therapy for those who show evident signs of the cited problems. Furthermore, interventions on work policies and personnel management aimed at workplace wellbeing and at reducing the risk of work-related stress problems are desirable.

It is apparent that LMDs are subjected to an enormous workload, to which must be added exhausting physical fatigue and the psychological and emotional burden of grappling with emergency, death, and illness on a daily basis. These are not new issues in Italy, to the medical community and to policy makers, and indeed over the years there have been attempts to deal with them, or at least to contain them, albeit with mixed results. It is essential that we better understand the barriers that still hinder the psychological and emotional wellbeing of LMDs, a problem that too often results in harm not only to the individual practitioner, but to the entire Italian national healthcare system. In our conclusions and operational proposals, we would like to highlight two important aspects and issues on which to base future developments. In choosing forensic medicine as a career, the forensic practitioner knowingly chooses a profession that requires a certain amount of personal sacrifice, as well as a great investment of time, attention, and personal peace of mind. However, LMDs, and workers in general, often feel unable to discuss illness, discomfort, suffering, or limitations that affect them due to the stigma associated with these conditions [55]. It will be necessary to plan pathways for them to learn to recognize their problems, discomfort, and suffering. Wellness increases as an individual takes more responsibility for their own health, including physical, mental, and emotional wellbeing [56]. The other aspect relates to the need to consider that LMDs are workers who, like all others, deserve healthy working conditions. Institutions cannot ignore the goodwill of their employees. Instead, they should face structural and systemic problems which require institutional diagnosis in order to better understand the pathologies affecting not the individual LMD but the organizational and relational system and the leadership that guides it and takes responsibility for it [57].

What follows is a summary of the most important steps outlined in this article:

i) Address institutional level problems by improving organizational support, effective communication with supervisors and colleagues, and implementing educational interventions to help employees recognize and relieve stress.
ii) Train managers and supervisors to recognize warning signs of stress-related symptoms in employees and initiate appropriate interventions.
iii) Implement training courses to encourage the development and use of functional coping strategies and discourage dysfunctional ones.
iv) Facilitate access to necessary mental health counselling and help for workers who are in need.
v) Address systemic stigma that prevents workers from speaking about their difficulties in the work context.

## Authors’ contributions

Ignazio Grattagliano, Sara Sablone and Matthew Groicher contributed to the research design, data curation and analysis, and article writing, and participated in the conception of the project, supervised the study and edited the article. Valeria Lagona and Maria Bellino carried out the data curation and wrote the article. Grazia M. Violante and Tamara Patrizia Fanco participated in the research design, data curation and article writing. Roberta Risola contributed to the conception of the project, and wrote the article.

## Compliance with ethical standards

Not applicable (literature review).

## Disclosure statement

The authors report there are no competing interests to declare.
